# Management of Late Preterm and Term Neonates exposed to maternal Chorioamnionitis

**DOI:** 10.1186/s12887-019-1650-0

**Published:** 2019-08-13

**Authors:** Mitali Sahni, María E. Franco-Fuenmayor, Karen Shattuck

**Affiliations:** 1 Division of Neonatology, Drexel University College of Medicine, Philadelphia, PA USA; 2 Department of Pediatrics, University of Texas Medical Branch, Galveston, TX USA; 3 Division of Neonatology, Department of Pediatrics, University of Texas Medical Branch, Galveston, TX USA; 4 St. Christopher’s Hospital for Children, 160 East Erie Avenue, Philadelphia, PA 19134 USA

**Keywords:** CBC, Newborn, Sepsis, Antibiotics

## Abstract

**Background:**

Chorioamnionitis is a significant risk factor for early-onset neonatal sepsis. However, empiric antibiotic treatment is unnecessary for most asymptomatic newborns exposed to maternal chorioamnionitis (MC). The purpose of this study is to report the outcomes of asymptomatic neonates ≥35 weeks gestational age (GA) exposed to MC, who were managed without routine antibiotic administration and were clinically monitored while following complete blood cell counts (CBCs).

**Methods:**

A retrospective chart review was performed on neonates with GA ≥ 35 weeks with MC during calendar year 2013. IT ratio (immature: total neutrophils) was considered suspicious if ≥0.3. The data were analyzed using independent sample T-tests.

**Results:**

Among the 275 neonates with MC, 36 received antibiotics for possible sepsis. Twenty-one were treated with antibiotics for > 48 h for clinical signs of infection; only one infant had a positive blood culture. All 21 became symptomatic prior to initiating antibiotics. Six showed worsening of IT ratio. Thus empiric antibiotic administration was safely avoided in 87% of neonates with MC. 81.5% of the neonates had follow-up appointments within a few days and at two weeks of age within the hospital system. There were no readmissions for suspected sepsis.

**Conclusions:**

In our patient population, using CBC indices and clinical observation to predict sepsis in neonates with MC appears safe and avoids the unnecessary use of antibiotics.

## Background

Chorioamnionitis or intraamniotic infection is defined as acute inflammation of the membranes and chorion of the placenta, typically due to ascending polymicrobial bacterial infection. Most commonly, chorioamnionitis is associated with ruptured membranes, but organisms including Group B streptococcus, Ureaplasma species, *Mycoplasma hominis,* and *Listeria monocytogenes* infect intact membranes as well. MC is a significant risk factor for early-onset sepsis (EOS) due to Group B streptococcus (GBS) and can reflect an intrauterine onset of infection in the neonate [1, 2]. The traditional diagnostic criteria for clinical chorioamnionitis include maternal fever (> 100.4 °F persisting more than 1 h or any fever more than 101 °F), and two or more of the following: fetal tachycardia (> 160/min), maternal tachycardia (> 100/min), uterine tenderness, and foul-smelling or purulent amniotic fluid.

To address possible EOS in asymptomatic newborns with MC, the 2010 American Academy of Pediatrics (AAP) and Center for Disease Control and Prevention (CDC) recommend a limited evaluation including blood culture (at birth) and CBC with differential (at birth and/or at 6–12 h of life (HOL) along with initiation of empiric antibiotics. However, a recent epidemiological study found that among term infants exposed to MC, only 13.5% received antibiotics after birth [3]. This deviation from guidelines, as seen in practice, has been attributed to improved obstetrical interventions, which significantly decreased the risk of sepsis in late preterm and term neonates in this setting [4]. A multispecialty workshop sponsored by the *Eunice Kennedy Shriver* National Institute of Child Health and Human Development, recommended treating late preterm and term neonates only if the diagnosis of chorioamnionitis is confirmed with pathology or microbiology [5]. The American College of Obstetricians and Gynecologists (ACOG) supported this and introduced the term intraamniotic infection for chorioamnionitis. They further defined suspected intraamniotic infection as maternal intrapartum fever (single maternal temperature ≥ 39.0 °C or a temperature of 38.0 °C–38.9 °C that persists for > 30 min during labor) presenting with 1 or more of the following: purulent cervical drainage, maternal leukocytosis, and fetal tachycardia [6]. The most recent AAP statement addressing management of neonates born at ≥35 weeks GA at risk for EOS suggests utilizing a locally tailored guideline by birth centers, for EOS risk assessment and clinical management [7]. They also suggested utilizing multivariate risk assessment tools like the Neonatal Early Onset Sepsis Risk Calculator to assess the risk of EOS [7–9]. In our hospital, the practice is to perform CBCs at 6 and 24 h after birth, and initiate the sepsis work-up and antibiotics (Ampicillin and Gentamicin) only if the baby develops signs/symptoms of sepsis or the CBCs suggest infection. Since our practice was initiated, others have looked at the management of neonates exposed to MC without antibiotics [10, 11]. The purpose of this study is to report our observational practice of managing asymptomatic neonates ≥35 weeks GA exposed to MC without empiric antibiotic administration. In addition, we include follow-up of these neonates after hospital discharge to evaluate the safety of our practice.

## Methods

A retrospective chart review was done on all neonates with GA ≥ 35 weeks, who were exposed to MC. The study included 275 neonates over 12 months (January to December 2013). The institutional review board at the University of Texas Medical Branch (UTMB) at Galveston approved this study. In November 2012, we instituted a practice guideline for management of asymptomatic neonates exposed to maternal chorioamnionitis, using serial CBCs and close observation. For the study, MC was defined as maternal treatment with antibiotics for chorioamnionitis. Data collected included the following:

- Neonatal demographic data included birth weight, gender, and gestational age.
- Maternal data including maternal age, GBS status and antibiotic treatment before delivery.
- Results of CBC with differential at 6 and 24 HOL, including the ratio of immature to mature neutrophils (I/T ratio), absolute neutrophil count (ANC), and total leukocyte count (TLC). The differential count is obtained manually by trained technicians and confirmed by hematologists. I/T ratio was considered suspicious if ≥0.3.
- Presence of signs and symptoms of sepsis including respiratory distress (tachypnea: respiratory rate > 60/min, increased work of breathing or need for oxygen), temperature instability (Temperature < 36.5 C that could not be attributed to environmental factors), hypoglycemia and feeding difficulty (poor feeding with low urine output).
- The incidence of sepsis work-up and transfer to neonatal intensive care unit (NICU).
- Results of blood culture and time interval for it, organisms isolated, length of antibiotic use and hospital stay.
- Chart review for follow up visit 2–3 days after discharge and at two weeks of age.
- Chart review for readmission after discharge to rule out sepsis.

The data collected were analyzed using descriptive statistics and independent sample T-tests. SPSS software platform was utilized to do the statistical analysis. *P* value < 0.05 was considered significant.

## Results

In 2013, 5006 babies ≥35 weeks gestation were born at UTMB. Two hundred seventy-five were born to mothers with a diagnosis of chorioamnionitis, for an incidence of 5.49%. At least two CBCs were obtained on all neonates with MC. The baseline characteristics of the study population are presented in Table 1. One hundred sixty (58.2%) of the infants were born via vaginal delivery. Of the 275 mothers with chorioamnionitis, the majority were not colonized with GBS. Of those who were positive for GBS, 90% were treated adequately. Table 2 illustrates the GBS status of the mothers and the information about their treatment. 68.4% of the mothers with the diagnosis of MC were administered antibiotics at least one hour before delivery.

**Table 1:** Baseline characteristics of the study population

Baseline characteristics of the study population	
Mean gestational age (weeks)	38.8 wks (range 35-41wks)
Birth weight (grams)	3368 g (range 2070–5029 g)
Male (%)	51.3
Infants born at ≥35 weeks gestation age (2013)	5006
Incidence of MC in infants ≥35 weeks gestation (%)	5.49

**Table 2:** GBS status of mothers with chorioamnionitis

GBS status	Percentage of mothers
Negative	84.4%
Positive- Adequately treated	10.2%
Positive- Not treated	1.1%
Unknown- Adequately treated	1.5%
Unknown- Not treated	2.9%

The first CBC was obtained at a mean of 5.85 HOL (range 0–12, mode 6). The mean ANC was 14.9 ± 5.5, and the mean I/T ratio was 0.29 ± 0.15 at this time. One hundred sixty-three infants (40.7%) had a suspicious I/T ratio (> 0.3) on the first CBC. Only 1 out of the 163 infants with increased I/T ratio at the time of first CBC was diagnosed with EOS with a positive blood culture. The positive predictive value (PPV) for early I/T ratio (> 0.3) to diagnose EOS was 0.61%(95% CI 0.56–0.68%). The second CBC was obtained at a mean of 22 HOL (range 7–32, mode 24). Mean ANC was 12.16 ± 4.8 and mean I/T ratio 0.18 ± 0.15. Fewer infants (18.54%) had a suspicious I/T ratio on the second CBC. The PPV of I/T ratio (> 0.3) to diagnose EOS at the time of the second CBC was 1.96% (95% CI 1.53–2.51%). In 48 infants the I/T ratio increased at the time of second CBC. This increase in IT ratio was not predictive of change in clinical presentation as only 6 out of these 48 infants had clinical signs of sepsis. Twenty-two of these infants had an initial CBC with I/T ≥ 0.3.

**Fig. 1:**
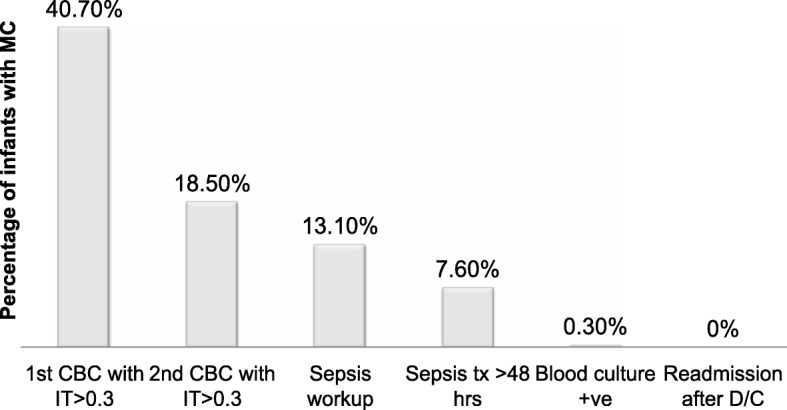
Progression of management of infants exposed to maternal chorioamnionitis

Not all neonates with an increase in I/T ratio were transferred to the NICU for further workup. The decision to transfer was made based on the overall clinical presentation of the infant and attending’s judgment. Thirty-six infants (13.1%) were transferred to the NICU for further evaluation including blood culture and antibiotics. Although only one infant had a positive blood culture, 21 infants (7.6%) were treated with antibiotics for more than 48 h due to concern for clinical sepsis (Fig. 1). The duration of antibiotics in these infants ranged from 3 to 7 days and was dependent on clinical judgment of the attending. The infant with a positive blood culture was born via vaginal delivery at 35 weeks GA and developed respiratory distress within the first 6 h after birth. She was transferred to the NICU due to clinical suspicion for EOS and was found to have a high I/T ratio of 0.72 and 0.48 at the time of the first and second CBC, respectively. She was treated with antibiotics for 10 days for culture positive sepsis. Infant’s mother was diagnosed with MC based on high maternal temperature (101 °F) along with fetal and maternal tachycardia. All neonates treated with > 48 h of antibiotics developed clinical signs of sepsis (respiratory distress, poor feeding or hypothermia) and 28% of these neonates had an increase in the I/T ratio. Figure 1 shows the percentage of infants with MC who received further management.

An independent-sample t-test was conducted to compare the CBC indices in the neonates who were treated with antibiotics for > 48 h with neonates who were either not treated or treated for ≤48 h (Table 3). Both IT ratios differed between the group treated with antibiotics > 48 h vs. those treated for ≤48 h and untreated group (*p* = 0.003 for the first CBC, *p* = 0.002 for the second CBC). At the time of first CBC, rupture of membrane (ROM) > 18 h was associated with a higher likelihood of a suspicious IT ratio (*p* = 0.023). There were no significant differences with regard to gender, birth weight, mode of delivery or presence of respiratory distress.

**Table 3:** ANC and IT ratio significance (*Mean ± S.D.)

Variables	Abx > 48 h	No Abx/ Abx ≤48 h	*P* value [95% CI for difference between means]
IT ratio* (1st CBC)	0.45 ± 0.24	0.27 ± 0.14	***0.003*** [0.07 to 0.29]
IT ratio* (2nd CBC)	0.38 ± 0.25	0.16 ± 0.13	***0.002*** [0.08 to 0.32]
ANC* (1st CBC)	10.55 ± 4.9	15.32 ± 5.4	***< 0.001*** [−7.1 to −2.4]
ANC* (2nd CBC)	14.05 ± 6.7	12.01 ± 4.6	0.197 [− 1.1 to 5.2]

Difference in CBC indices between the groups treated with prolonged antibiotics for a diagnosis of clinical sepsis vs. the group not treated with antibiotics and treated for 48 h or less. ANC: absolute neutrophil count; Abx: antibiotics; Significant *p* values are presented in bold

All newborns are scheduled for follow-up appointments at 2–3 days and 2-weeks after discharge. Two hundred twenty-four neonates (81.5%) attended the 2–3 days appointment and 226 (82.2%) were seen at two weeks of age at a clinic within our hospital system where a full history and physical exam was obtained. At the time of follow up visits, none of these infants exposed to maternal chorioamnionitis demonstrated any concern for sepsis. Furthermore, none of the infants in our study were readmitted to our hospital for evaluation of sepsis.

## Discussion

Maternal chorioamnionitis is a well-known risk factor for EOS; however, there is considerable variation in the accepted definition and diagnosis of MC. Frequently it is used to describe a heterogeneous group of conditions ranging from sterile inflammation to infection of varying degree of severity and duration. Some centers diagnose MC by the presence of intrapartum fever alone (unexplained by other infection), while others favor a diagnosis described by Gibbs et al. [12] that requires presence of intrapartum fever of at least 100 °F (37.8 °C) as well as at least two additional clinical signs (maternal tachycardia > 100 beats per minute, fetal tachycardia ≥ 160 beats per minute), uterine tenderness, foul-smelling amniotic fluid, and leukocytosis ≥ 15,000 cells/mm^3^) [13]. There are other variations of this definition also in use, and hence the term chorioamnionitis does not consistently reflect the severity of maternal and fetal illness [14]. In our setting, the obstetricians assess the risk for chorioamnionitis using the clinical criteria proposed by Gibbs et al. described above. Antibiotics are begun only if they make the clinical diagnosis. Our setting is unusual in that the obstetricians (all in the academic maternal-fetal medicine group) have a unified approach to the diagnosis and treatment. The babies born to those mothers are the subject of this report. We do not have a histological diagnosis for these cases.

The 2010 guidelines by the CDC [15] and AAP [16] recommend treatment of all neonates exposed to MC irrespective of gestational age and presence of symptoms. As a consequence of these guidelines, many term and late preterm neonates are unnecessarily exposed to antimicrobial agents in order to prevent rare cases of EOS. Surveillance studies estimate the incidence of EOS to be 0.77 cases/1000 live births. This can be further broken down to ~ 0.5 cases/1000 among those born at ≥37 weeks, compared to ~ 3.0 cases/1000 live births occurring at < 37 weeks gestation [17]. However, the presence of chorioamnionitis increases this risk by 2–3 fold [18]. Reported rates of confirmed EOS in infants born at ≥35 weeks’ gestation to mothers with clinical chorioamnionitis range from 0.47 to 1.24% [4, 14]. This risk of EOS is reduced if symptomatic newborns are excluded from the analysis [4, 19]. In a multicenter, prospective surveillance study EOS was diagnosed in 389 of the 396,586 infants reviewed; 60% of these infants were exposed to MC. The researchers estimated that almost 450 term infants exposed to MC would have to be treated for every case of confirmed EOS [20]. Despite being a relatively small sample size, the data from our study is consistent with the observation that the risk of EOS is low in infant exposed to MC (1 case of EOS in 275 infants, 0.36%). There were no cases of EOS in infants that were exposed to MC who remained asymptomatic within the first 24 h. Babies who were < 35 weeks or were clinically ill at birth were admitted directly to the NICU and are not addressed in this report.

A multivariate risk assessment model to predict the risk of EOS, was developed using objective data collected at the time of birth [21] and the evolving newborn condition during the first 6 to 12 h after birth [22]. The objective data included the highest maternal intrapartum temperature, the maternal GBS colonization status, the type and duration of intrapartum antibiotic therapies, the duration of ROM and gestational age at the time of delivery. One web-based Neonatal Early-Onset Sepsis Risk Calculator (https://neonatalsepsiscalculator.kaiserpermanente.org) uses a predictive model developed from a large population to calculate the risk estimate for EOS and recommend management [23]. The recent AAP statement regarding management of suspected/proven EOS in neonates ≥35 weeks’ gestation suggests utilizing the sepsis calculator in conjunction with serial physical exams every 4–6 h for the first 48 h [7]. At our center, we continue to use serial physical exams and clinical assessment to evaluate for risk of EOS in asymptomatic term infants. Use of multivariate risk assessment tool has not been implemented in practice.

In 2016, an Expert Panel Workshop summary by the ACOG questioned the current method of diagnosing chorioamnionitis and proposed the use of an alternate diagnosis of Triple I for pathological diagnosis of chorioamnionitis. They further recommended treating late preterm and term neonates only if the diagnosis of chorioamnionitis is confirmed with pathology or microbiology. This would involve the presence of clinical symptoms and any one of the following:

1. Positive gram stain on amniocentesis;
2. Low glucose or positive amniotic fluid culture; or
3. Placental pathology revealing infection [5].

However, this information is rarely available to use at the time of birth when the decision to do a sepsis workup is being made. In August 2017, the ACOG Committee on Obstetric Practice released a committee opinion statement about evaluation and management of intrauterine infection [6], which clarified their earlier recommendations and suggested the use of multivariate risk assessment and increased reliance on clinical observation to safely decrease the number of well-appearing term newborns being treated empirically with antibiotics. However, they did not include Triple I as a new diagnostic category.

Antibiotic therapy in late preterm and term neonates born to mothers with MC ideally should be restricted to those who are at increased risk of developing EOS. Unfortunately, no diagnostic test effectively predicts the infants at risk. Hence there are different approaches used by different centers to limit the unnecessary use of antibiotics in this setting. Some centers use C-reactive protein in addition to CBC indices to predict the risk of EOS [10, 19, 24] and some use the neonatal EOS calculator to help with management of these neonates [9, 11]. Most neonates at UTMB are born to mothers who receive prenatal care in a system of Regional Maternal and Children’s clinics, which is staffed by members of the OB faculty. Thus, we have the advantage of large delivery service with a consistent practice of screening for GBS and a standard definition of MC. At our center, we perform a sepsis evaluation on the basis of CBC indices and presence of other risk factors including duration of rupture of membranes, the severity of maternal illness and maternal GBS colonization.

Historically, CBC indices such as WBC count, ANC, IT ratio and platelet counts have been used to predict the risk of EOS. Hornik et al. studied 166,092 infants and found that low WBC count, low ANC, and high IT ratios were associated with increased odds of infection (highest odds ratios: 5.38, 6.84, and 7.97, respectively). They also found that the specificity and negative predictive values of using CBC indices were high (73.7–99.9 and > 99.8%, respectively). However, sensitivities were low (0.3–54.5%) for all complete blood cell count indices analyzed [25]. Based on these results they recommend against the sole use of CBC indices to rule out infection, but to continue monitoring for clinical signs of sepsis and maintaining a high index of suspicion for those infants at risk remains necessary. Another large retrospective cross-sectional study evaluated CBC indices for predicting the risk of sepsis and found that WBC count, ANC and IT ratio were better at discriminating the risk of infection after 4 h of birth as these indices can be affected by many other factors in the first few hours of birth [26]. Hence, as part of our clinical protocol, we obtain the first CBC at about 6 h of life in asymptomatic neonates. In the study by Hornik et al., elevated I/T ratios (> 0.2, > 0.25, > 0.5) were associated with relatively high specificities (73.7, 81.7, 95.7%, respectively) and negative predictive values (99.2, 99.2, 99.0%, respectively) [25] to predict the risk of EOS. In our study, at the time of first CBC, the lowest value of IT ratio was 0.19. The higher IT ratio seen in our population may be attributed to inflammatory response associated with chorioamnionitis and frequently associated risk of prolonged ROM. Our analysis showed that I/T ratio > 0.3 had a poor positive predictive value for diagnosis of EOS (0.61 and 1.96% at the time of the first and second CBC respectively). I/T ratios were not reliably predictive of the perceived need for antibiotic treatment, with a sensitivity of only 28% for infants with that outcome. Therefore our data supports the recommendation to not use CBC indices alone, to predict the risk of EOS.

Our study has some limitations. First, our sample size was relatively small. Since the incidence of EOS is relatively low, our study may not be powered to estimate the risk of not treating the neonates with MC. Second, clinical sepsis was treated for more than 48 h based on clinical presentation in most cases. Third, our follow up data was limited to 81.6% of neonates in the study, and cannot predict if any of those with lack of follow up within our system were admitted elsewhere for EOS. However, our center is the most common pediatric provider for babies born at UTMB, due to the characteristics of the population we serve.

## Conclusions

In our population, we chose to monitor clinical status and CBC indices in neonates exposed to MC to avoid NICU admission, prolonged length of stay, separation from mothers, and antibiotics in a large group of neonates who would prove to be healthy. CBC indices should not be used alone to predict the risk of EOS. This study adds evidence to support the use of observation-based approaches for managing newborns exposed to maternal chorioamnionitis. Using clinical observation and laboratory evaluation, we were able to avoid these adverse events in 87% (239/275) of neonates exposed to MC. Our follow up data from neonates after discharge attests to the safety of observation-based approach for the management of maternal chorioamnionitis. Although this study is insufficiently powered to assure complete elimination of EOS, the current recommendations for close follow-up after discharge is a useful safety net.

## Data Availability

The datasets used and/or analyzed during the current study are available from the corresponding author upon reasonable request.

## Abbreviations

AAP: American Academy of Pediatrics
ACOG: American College of Obstetricians and Gynecologists
ANC: absolute neutrophil count
CBC: complete blood cell count
CDC: Center for Disease Control and Prevention
EOS: early-onset sepsis
GA: gestational age
GBS: Group B Streptococcus
HOL: hours of life
MC: maternal chorioamnionitis
NICU: neonatal intensive care unit
ROM: rupture of membrane
TLC: total leukocyte count
UTMB: University of Texas Medical Branch
